# Altered brain connectivity in Long Covid during cognitive exertion: a pilot study

**DOI:** 10.3389/fnins.2023.1182607

**Published:** 2023-06-22

**Authors:** Leighton Barnden, Kiran Thapaliya, Natalie Eaton-Fitch, Markus Barth, Sonya Marshall-Gradisnik

**Affiliations:** ^1^National Centre for Neuroimmunology and Emerging Diseases, Menzies Health Institute Queensland, Griffith University, Southport, QLD, Australia; ^2^Centre for Advanced Imaging, The University of Queensland, Brisbane, QLD, Australia; ^3^School of Information Technology and Electrical Engineering, The University of Queensland, Brisbane, QLD, Australia

**Keywords:** Long Covid, fMRI, connectivity, brainstem, medulla, midbrain, upregulation

## Abstract

**Introduction:**

Debilitating Long-Covid symptoms occur frequently after SARS-COVID-19 infection.

**Methods:**

Functional MRI was acquired in 10 Long Covid (LCov) and 13 healthy controls (HC) with a 7 Tesla scanner during a cognitive (Stroop color-word) task. BOLD time series were computed for 7 salience and 4 default-mode network hubs, 2 hippocampus and 7 brainstem regions (ROIs). Connectivity was characterized by the correlation coefficient between each pair of ROI BOLD time series. We tested for HC versus LCov differences in connectivity between each pair of the 20 regions (ROI-to-ROI) and between each ROI and the rest of the brain (ROI-to-voxel). For LCov, we also performed regressions of ROI-to-ROI connectivity with clinical scores.

**Results:**

Two ROI-to-ROI connectivities differed between HC and LCov. Both involved the brainstem rostral medulla, one connection to the midbrain, another to a DM network hub. Both were stronger in LCov than HC. ROI-to-voxel analysis detected multiple other regions where LCov connectivity differed from HC located in all major lobes. Most, but not all connections, were weaker in LCov than HC. LCov, but not HC connectivity, was correlated with clinical scores for disability and autonomic function and involved brainstem ROI.

**Discussion:**

Multiple connectivity differences and clinical correlations involved brainstem ROIs. Stronger connectivity in LCov between the medulla and midbrain may reflect a compensatory response. This brainstem circuit regulates cortical arousal, autonomic function and the sleep–wake cycle. In contrast, this circuit exhibited weaker connectivity in ME/CFS. LCov connectivity regressions with disability and autonomic scores were consistent with altered brainstem connectivity in LCov.

## 1. Introduction

Subjects suffering ‘Ongoing Symptomatic COVID-19’ (National Institute for Health and Care Excellence, 2020) complain of persisting fatigue, shortness of breath, and cognitive dysfunction (Soriano et al., 2022). In common with much of the literature we here label this condition long-COVID (LCov). The symptom characteristics of long-COVID resemble those of myalgic encephalomyelitis/chronic fatigue syndrome (ME/CFS) (Wong and Weitzer, 2021) and it has been suggested that LCov symptomatology is consistent with brainstem dysfunction (Yong, 2021).

While we were acquiring advanced brain MRI data for a new ME/CFS study we took the opportunity to select a sample of LCov subjects, using criteria in ME/CFS questionnaires, to perform the same MRI protocol.

Quantitative brain MRI studies in long-Covid are sparse, especially studies designed to detect differences in brain connectivity which underlies normal behavior and cognition. Functional MRI (fMRI) acquires several hundred MRI images that are blood oxygen level dependent (BOLD), either during resting state or a cognitive task. Correlations between the BOLD time series from different locations characterize connectivity between them. The most basic analysis derives connectivity between pairs of *a-priori* regions-of-interest (ROIs), e.g., hubs of intrinsic networks, and compares patient and healthy control (HC) populations. The risk of missing affected brain locations with limited *a-priori* ROIs can be mitigated by also computing connectivity between each ROI and all voxels in the rest of the brain to locate clusters of voxels where connectivity differs from HC. Further insights can be gained by seeking correlations between connectivity and disease severity. Sensitivity to changes in brain function is enhanced by acquiring fMRI while the subject is performing a cognitive task.

In ME/CFS, fMRI studies have been performed with disparate methodology to localize regions with altered connectivity. Hubs of brain intrinsic networks were a natural focus. The tri-network model proposed by Menon (2011) integrates 3 key intrinsic brain networks — the central executive network (CEN), salience network (SA), and the default mode network (DM), into a single cohesive model underlying normal behavior and cognition. This model predicts that the levels of *internally*-directed cognition of the DM and *externally*-directed cognition of the CEN networks are anti-correlated and under the control of the SA, such that if the brain engages one of the two, the SA inhibits the activation of the other (Shaw et al., 2021). Such a reciprocal relationship implies that if SA to DMN connectivity is different to HC in LCov, then SA to CEN connectivity will also differ (with opposite sign).

Most ME/CFS fMRI studies analyzed resting state fMRI and reported varied results mostly involving hubs of the salience (SA) and default mode (DM) networks (Boissoneault et al., 2016; Gay et al., 2016; Wortinger et al., 2017; Manca et al., 2021), although altered SA right insula connectivity was a finding in all studies. Two fMRI studies have been performed during a cognitive task. The first (Baraniuk et al., 2022) examined only the BOLD signal amplitude and reported diminished BOLD in the midbrain (before physical exercise). The second (Shan et al., 2018a; Barnden et al., 2019; Su et al., 2022) evaluated BOLD correlations (connectivity) between the brain’s default mode (DM) and salience (SA) network hubs and hippocampal and brainstem nuclei and detected altered DM connectivity (Shan et al., 2018b), weaker connectivity within the brainstem (Barnden et al., 2019) and altered connectivity between SA and DMN hubs (Su et al., 2022). Compromised SA and brainstem regulatory function will have consequences for cognitive and autonomic function (Hall, 2011) and leads to our hypothesis, based on overlapping symptoms, that LCov would also exhibit altered intrinsic brain connectivity.

Here we test the hypothesis that intrinsic brain connectivity in LCov is different to HC. For 10 LCov and 13 HC subjects fMRI was acquired on a 7T research MRI scanner which yields superior quality (signal to noise ratio) images relative to conventional 3T scanners, particularly in the brainstem (Colizoli et al., 2020). Patient selection and data analysis was informed by our experience with ME/CFS which shares many LCov symptoms. During the scan, subjects performed the Stroop color-word cognitive task which challenges conflict monitoring/cognitive control between task relevant and task-irrelevant stimuli (Egner and Hirsch, 2005).

## 2. Methods and materials

### 2.1. Subjects

The study was approved by Griffith University Human Research Ethics Committee (2022/666) and written informed consent was obtained from all individuals. This cross-sectional investigation was conducted at the National Centre for Neuroimmunology and Emerging Diseases (NCNED) on the Gold Coast, Queensland, Australia. Eligible participants were medical-practitioner referred and assessed using the NCNED research questionnaire for fatigue affected subjects, which recorded severity of post-exertional malaise, cognitive disturbances, immune manifestations, thermoregulatory complaints, gastrointestinal symptoms, urinary frequency, body pain, and sleep disturbances. Long-COVID participants were selected with one or more of these symptoms, with moderate or worse severity, with onset less than 3 months following COVID-19 infection and persisting for at least 3 months, according to the WHO working case definition (Soriano et al., 2022). Healthy controls reported no chronic health condition or evidence of underlying illness. Participants were aged between 18- and 65-years. Medical history was requested to identify comorbid manifestations or exclusionary diagnoses including mental illness, malignancies, autoimmune, neurological, or cardiovascular diseases. Female participants were not pregnant or breastfeeding. Finally, 10 Long-COVID (7F, 3M) who met the WHO clinical case definition and 13 healthy control subjects (7F, 6M) were included in this study.

### 2.2. MRI acquisition

Two 7.5 min fMRI acquisitions separated by 90 s were acquired sagitally for each subject during the cognitive task. In this work we confine ourselves to analysis of the first fMRI acquisition. The fMRI image volumes were acquired on a 7T whole-body MRI research scanner (Siemens Healthcare, Erlangen, Germany) with a 32-channel head coil (Nova Medical Wilmington, United States). For each fMRI, 225 volumes of fMRI data were acquired using a multiband echo-planar imaging (EPI) pulse sequence developed at the University of Minnesota (Auerbach et al., 2013), with 80 sagittal slices, multiband factor = 3, TR = 2000 ms, TE = 22.4 ms, flip angle = 70°, acquisition matrix 192 × 192 and voxel size 1.25 mm^3^. The 225 fMRI volumes were acquired while the subject responded to a sequence of Stroop color-word tests (Leung et al., 2000).

An anatomical image was acquired using Siemens T2 ‘SPACE’ optimized 3D fast spin-echo (T2wSE) TR = 3200 ms, TE = 563 ms, scans on an adjacent 3T scanner on the same day. Acquisition time was 5:44 (min:sec). These scans employed an optimized variable flip angle sequence (Siemens SPACE) to yield a ‘true 3D’ acquisition in a shortened time. Their ‘contrast equivalent’ TE compares with standard T2wSE TE (Busse et al., 2006) although the signal is also influenced by T1 relaxation (Mugler, 2014), possibly more than for T2wSE. These T2 ‘SPACE’ images were sagittal with pixel size 0.88 × 0.88 × 0.9 mm. T2 ‘SPACE’ were chosen for the anatomical scan because their spin-echo sequence renders them resistant to the magnetic field induced distortions of the conventional gradient-echo MPRAGE sequences (Bushberg et al., 2002), particularly in the brainstem.

### 2.3. Cardiac and respiratory monitoring

Physiological noise in the fMRI BOLD time series was modeled using 200 Hz time-series from pulse oximeter and respiratory strap sensors recorded during the scan. The cardiac and respiratory time series interactions were computed using the TAPAS toolbox (Kasper et al., 2017) and used as covariates to correct physiological noise.

### 2.4. The Stroop task

For each task fMRI, the Stroop task was used to investigate the attention and concentration difficulties reported by Long COVID patients (Leung et al., 2000). Each Stroop task displayed two colored words. The upper word, either RED, BLUE, YELLOW, or XXXX, was colored either red, blue, or yellow on a black background. The lower word was either RED, BLUE, or YELLOW colored white on a black background. The subject was asked to decide whether the *color* of the upper word agreed with the *meaning* of the lower word and press one of two buttons on a handpiece to respond ‘yes’ or ‘no.’ The Stroop task fMRI was divided into four conditions, three were trials: either *neutral* (upper word is XXXX), *incongruent* (the upper word is in a color different to the lower word meaning), or *congruent* (upper word is in the color of the lower word meaning). The incongruent task is considered more challenging because of the inhibitory element required to overcome the natural impulse to read the top word rather than only inspect its color, and so decide meaning vs. meaning of the lower word rather than color vs. meaning. The fourth condition, *rest*, was the period between a trial response and the next trial onset. During this ‘rest’ condition a fixed stationary cross appeared on the screen for a period randomized between 3 and 12 s.

In each task fMRI, a total of 60 Stroop trials were randomly distributed over each 7.5 min acquisition with average inter-stimulus time of 10.5 s. 40% of trials were incongruent, 30% were congruent, and 30% were neutral. For each trial type, the stimulus-onset and response times were recorded. Their difference was reaction time. For each trial type, mean reaction time and accuracy = correct/total responses were evaluated for each fMRI run for each group.

### 2.5. Clinical scores

Clinical Scores used in regressions with LCov connectivity were SF36physical, SF36mental, Disability, Life (domestic) Activity, Heart rate (HR) and Heart rate Variability (HRV) and Respiration Rate (Resp) and Variability (RespV). HR, HRV, Resp and RespV were extracted from the power spectra of the pulse oximeter and respiration strap time series recorded during the 7.5 min scan (peak frequency and full width of peak at half maximum). Symptom scores were extracted from the Research Registry questionnaire developed by NCNED with the Centres for Disease Control and Prevention (CDC) and accessed online through Lime survey. Symptom severity was scored on a 0–5 scale (0: none, 1:very mild, 2:mild, 3: moderate, 4:severe, 5: very severe). The SF36 scores (Ware et al., 1995) were the average of Physical Functioning (10 items) and Mental Health (5 items) domains. Disability was the Bell Disability scale (Bell, 1995) and Life Activities was from the WHODAS assessment (Ustun et al., 2010).

### 2.6. Regions of interest

Table 1 lists the 20 MNI space ROIs that were assessed: 7 in SA hubs, 4 in DMN hubs, 5 reticular activation system (RAS) nuclei, periacqueductal gray matter (PAG) in the midbrain, the left culmen of cerebellum, and bilateral hippocampus nuclei. SA and DMN regions were supplied with the CONN package (Whitfield-Gabrieli and Nieto-Castanon, 2012). The 9 subcortical regions outside of the SA and DMN were selected because of their involvement in ME/CFS reported in an earlier MRI study (Barnden et al., 2016) or their rich connections (hippocampus subiculum) to brainstem RAS nuclei (Edlow et al., 2016). See (Barnden et al., 2019) for details of subcortical ROI creation.

**Table 1:** The 20 ROIs between which connectivity was assessed.

ROI location	Laterality	Abbreviation	Voxels
Salience network (7)			
Anterior insula	L&R	SA.AI_L, SA.AI_R	446, 388
Supramarginal gyrus (BA40)	L&R	SA.SMG_L, SA.SMG_R	233, 284
Rostral prefrontal cortex	L&R	SA.rPFC_L, SA.rPFC_R	1166, 581
Anterior cingulate cortex	Midline	SA.ACC	1063
Default mode network (4)			
Inferior lateral parietal	L&R	DM.LP_L, DM.LP_R	1041, 1326
Medial prefrontal cortex	Midline	DM.mPFC	1346
Posterior cingulate cortex	Midline	DM.PCC	4833
Brainstem (6)			
Rostral medulla	L&R	Mdul_L, Mdul_R	123
Midbrain: cuneiform nucleus	L&R	CnF_L, CnF_R	125
Midbrain: dorsal raphe nucleus	Midline	DoRph	129
Midbrain: periaqueductal gray	Midline	PAG	66
Subcortical (3)			
Culmen of cerebellum	L	Culm_L	123
Hippocampus: subiculum	L&R	Hippo_L, Hippo_R	377, 377

Salience (SA) and default mode (DM) network hubs were provided in the CONN package. L is Left, R is Right. Voxel volume was 1.95 mm^3^.

### 2.7. MRI processing

The fMRI data was pre-processed using the default preprocessing pipeline in the CONN toolbox (Whitfield-Gabrieli and Nieto-Castanon, 2012; Nieto-Castanon, 2020) dedicated to connectivity evaluation and statistical comparisons. Motion was computed during the series of 225 volumes. After omitting the first 5 volumes while the magnetic field reached a steady state, the average of the motion corrected volumes was spatially normalized to a template image in MNI space and the deformation applied to all motion corrected volumes. The 1.25 mm voxel size of the original 7T fMRI was retained throughout. The anatomical image was segmented into gray matter, white matter, and CSF for calculation of their volumes for use as denoising covariates. See the CONN handbook (Nieto-Castanon, 2020) for details. Time series of the 6 rigid-body motion parameters, cardiac and respiratory time series and their interactions, and a binary covariate identifying outlier intra-fMRI movements were used in denoising of fMRI mean ROI BOLD or voxel BOLD time-series.

For each fMRI voxel, BOLD time-series were computed. ROI time series were the average of their voxel time series. Denoising of the ROI (and voxel) BOLD time-series incorporated physiological noise, motion parameters, and total white matter and CSF volumes as nuisance covariates. After denoising, a temporal bandpass filter was used to remove frequencies from the BOLD time series below 0.008 Hz and above 0.15 Hz (Sasai et al., 2021).

For each subject, after denoising and filtering the BOLD time-series for each voxel, the mean BOLD time-series in each of the 20 ROIs (seeds) was computed. For each pair of ROIs, correlation between their BOLD time-series yielded a correlation coefficient, used here to characterize their functional connectivity (FC). Differences between FC of the 13 HC and 10 LCov were tested using CONN’s ROI-to-ROI approach. Regressions between the 10 LCov FC and clinical measures were also tested. Then for each ROI, its mean BOLD time series was correlated with the BOLD time series for each voxel in the brain using CONN’s ‘Seed-to-voxel’ utility to yield a 3D image of correlation coefficients. For each voxel a test for ROI connectivity differences between the 13 HC and 10 LCov was performed and then significant voxel clusters were reported. Clusters were defined by the conventional *uncorrected* p threshold of 0.001, and because we retained a very small voxel size (1.25 mm compared to the conventional 2 mm) with higher noise levels we also assessed clusters defined by an *uncorrected* 0.005 threshold. We reported clusters with p-FDR < 0.05. Functional Connectivity values were computed for the entire set of 180 connection pairs between the 20 ROIs. The terms connectivity and ‘correlation coefficient’ are used interchangeably here.

### 2.8. General linear model

To compare the LCov and HC groups, ‘second-level’ CONN analysis applied a multivariate parametric General Linear Model (GLM) for all correlation coefficients (connectivity values). Age was included as a covariate. The GLM model here was


$$Y_{i}=\beta_{1}.X_{\mathrm{i1}}+\beta_{2}.X_{\mathrm{i2}}+\beta_{3}.X_{\mathrm{i3}}+\varepsilon_{i}$$

where, for a given pair of ROIs (seeds), Y_i_ are the FC connectivity values (i = 1 to 23 subjects), X_i1_, X_i2_, are binary vectors that specify group membership (for X_i1_ 1 is HC, 0 is LCov; for X_i2_ 0 is HC and 1 is LCov). X_i3_ is the vector of ages for the 23 subjects. Solving the GLM equation yields β_1_, β_2_ and β_3_ the relative contribution of group and age to the observed variation in Y_i_. ε_i_ is the residual variation not explained by group membership or age, which has zero mean and variance σ^2^, and is used to estimate statistical inference of β_1_ and β_2_. Group differences were tested with contrast [1–1 0].

For the seed-to-voxel analysis, Y_i_ is the vector of connectivity *images* for each subject. That is, for a particular ROI, Y_i_ is the 3D image in which each voxel value is the correlation coefficient between the ROI mean BOLD time-series and that voxel’s BOLD time-series. The significance of clusters of voxels was derived using random field theory (Friston et al., 1994). Clusters with *p* < 0.05 are reported here with locations from CONN’s internal atlas.

A similar model was used to test for correlations between LCov ROI-to-ROI connectivity and LCov clinical scores, with X_i1_ for LCov group membership, X_i2_ for LCov clinical score and X_i3_ for age, and contrast [0 1 0].

### 2.9. Statistical inference

Results were reported by CONN as the T statistic for connectivity difference for each ROI pair and a false discovery rate (FDR) corrected value of *p* (Benjamini and Hochberg, 1995) defined as the expected proportion of false discoveries among all ROI pairs with similar or larger effects across the entire set of 180 pairs. Each ROI pair with an FDR corrected value of *p* <0.05 for difference between LCov and HC is reported below.

The seed-to-voxel tests for significant clusters were 1-sided with clusters defined by an uncorrected threshold voxel P (uvP) = 0.001 and 0.005. Clusters with p-FDR < 0.05 are reported here.

## 3. Results

### 3.1. Subject symptoms

The symptoms scored by each of the 10 LCov subjects and their age, gender and severities are given in Table 2. All 10 subjects reported moderate to very severe fatigue, muscle aches and unrefreshing and/or disturbed sleep. 7/10 subjects reported cognitive (concentration and/or memory) problems. Age mean (standard deviation) was 44 (15) for LCov and 39 (13) for HC. Gender was F/M = 7/3.

**Table 2:** Symptoms (left column) that were scored by each subject, and subject gender, age and symptom severity scores.

Subject	1	2	3	4	5	6	7	8	9	10
Gender	F	M	F	F	M	F	F	M	F	F
Age	49	64	46	30	40	59	43	61	32	19
Fatigue	4	3	3	3	3	3	4	4	4	3
Concentration	3	3	3		4	3	4	4		
Memory					3		4	3		
Muscle aches	4	3	3	4	4	3	3	4	3	3
Joint pain	3	3	3			3	3			4
Headaches	3	3			4	3	3	3	3	3
Sensitivity to light	3	3			4	3	3			
Sensitivity to noise/vibration	3	3								
Odor/taste							3	3		
Unrefreshing sleep	3	4	3		4	3	4	3	5	4
Sleep disturbances	3	3	3	3	4		3	3		5
Muscle weakness				4		4	3	4		
Lymph						4	3	4	3	
Throat										4
Fever							5	3		
Nausea					3		5			5
Abdominal pain	3				3		5		3	5
Bloating		3		3			4		4	4
Bowel							5			4
Urinary frequency										4
Breathing	3			3			4			
Light headed	3		3		4				3	3
Arrythmia										4
Sweating							4	3		
Cold extremities				3			4		3	

Severity scores relevant to LCov selection were 3: moderate, 4: severe, 5: very severe.

### 3.2. Stroop task accuracies and reaction times

Table 3 shows that, for the three categories of the Stroop task, LCov accuracies were lower than for HC, although the difference was only significant (*p* = 0.026) for the Neutral task. Similarly, LCov Reaction Times were slower than HC but were not significant (*p* > 0.05).

**Table 3:** Healthy control (HC) and long Covid (LCov), Stroop task accuracies and reaction times for each of the three categories of Stroop task (incongruent, congruent and neutral).

Cohort	*N*	Accuracy			Reaction time (seconds)			Stroop
		Incongruent	Congruent	Neutral	Incongruent	Congruent	Neutral	Effect
HC	13	0.940	0.982	0.995	1.413	1.221	1.230	0.139
LCov	10	0.923	0.973	0.901*	2.094	1.826	1.685	0.162

Stroop Effect = (Incongruent Reaction Time – Congruent Reaction Time) / Mean Reaction Time.

*Significantly lower than HC (*p* = 0.026).

### 3.3. LCov vs. HC connectivity

**Figure 1:**
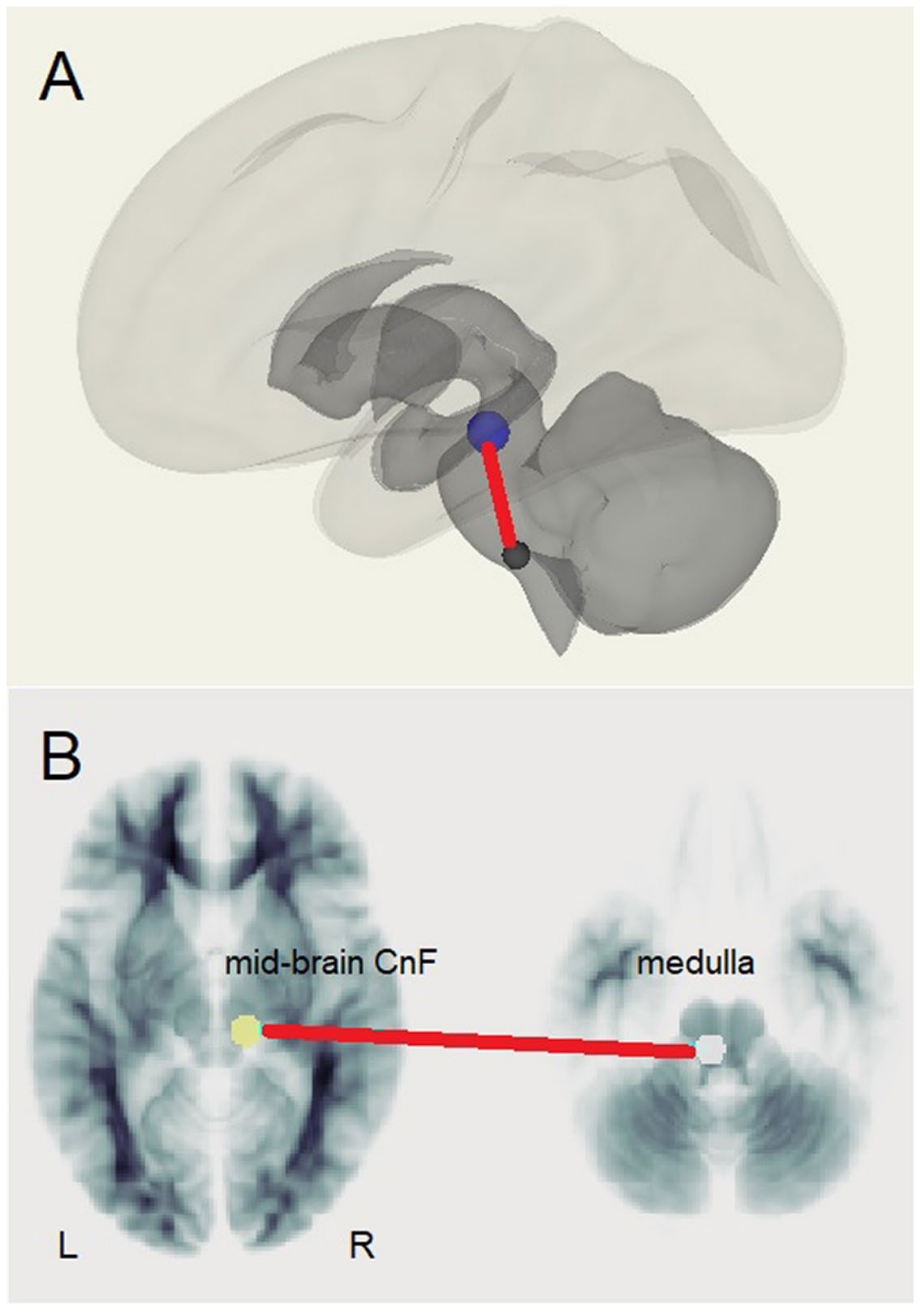
The brainstem connection between the midbrain right cuneiform nucleus and left rostral medulla that was stronger in LCov than HC. **(A)** Medulla (lower) to Cuneiform Nucleus (upper) connection shown relative to surface images of subcortical (dark) and cortical (light) white matter. **(B)** Left medulla and right cuneiform nucleus (CnF) shown on reference white matter transaxial sections.

**Figure 2:**
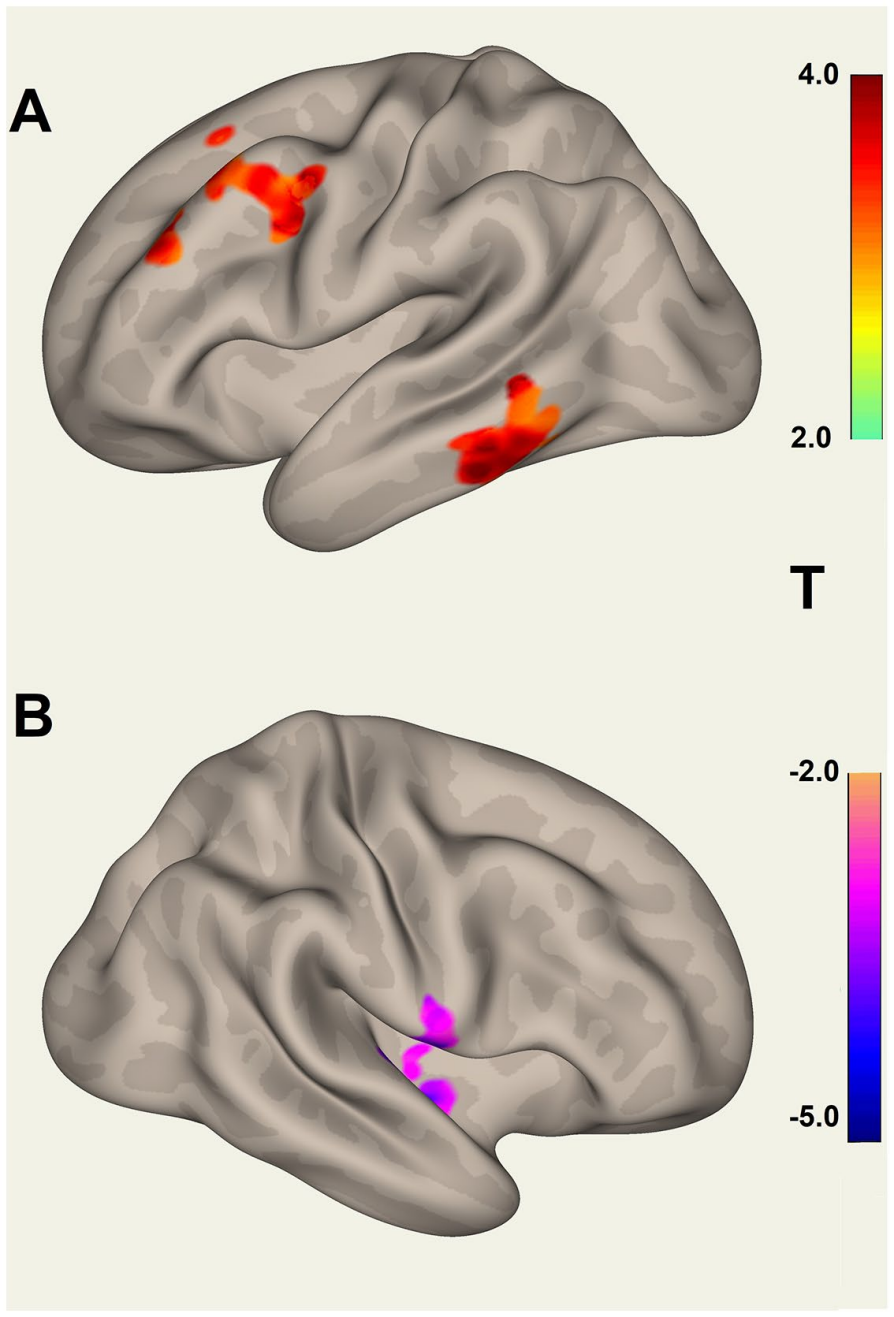
Voxel clusters that connect to the Salience Network left Anterior Insula seed, where connectivity for HC differs from LCov. **(A)** Two clusters for HC > LCov connectivity in left inferior temporal area (lower) and left middle frontal and pre-central areas (upper), and **(B)** for HC < LCov connectivity in the right central operculum. The clusters are contiguous above and/or beneath the reference white matter surface. Refer to Table 5 (SA.AI_L) for details.

**Figure 3:**
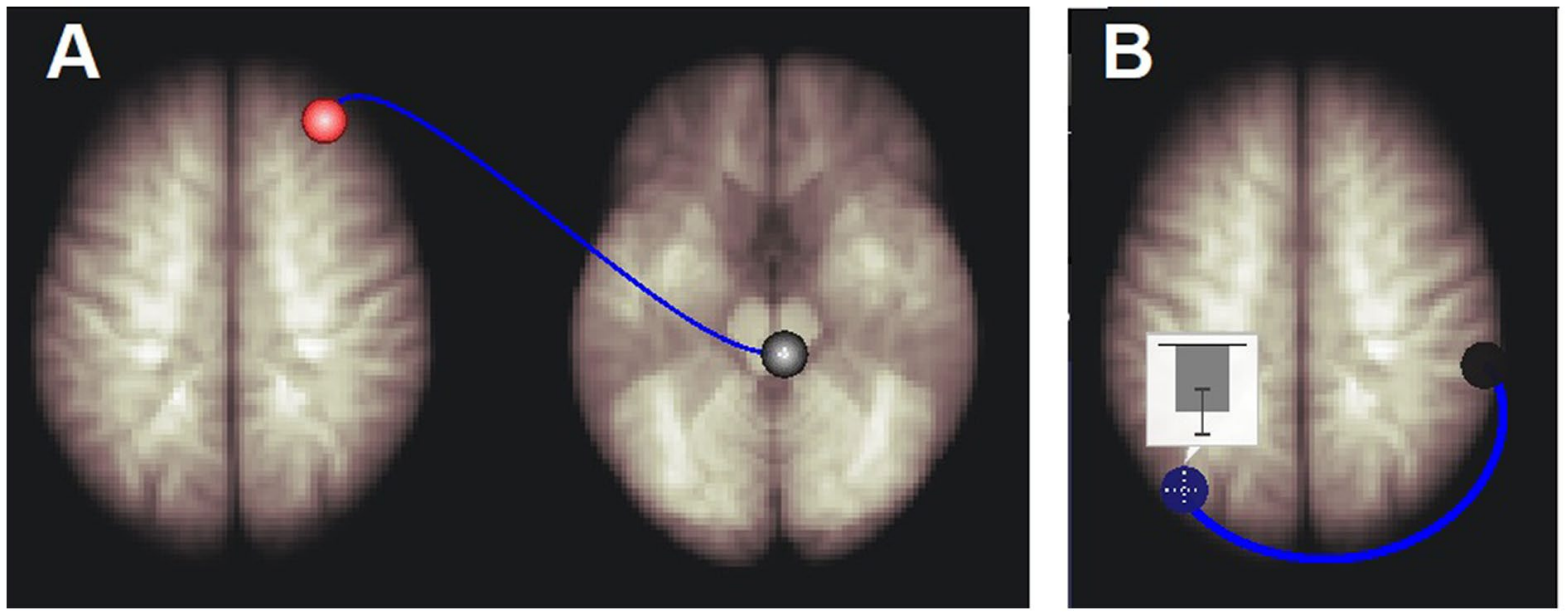
Long Covid connections that correlated with respiration rate variability. **(A)** Right medulla to right rostral prefrontal cortex connectivity was positively correlated with respiration rate variability. **(B)** Left default mode network inferior lateral parietal hub (DM.LP_L) to right salience network supra marginal gyrus hub (SA.SMG_R) connectivity was negatively correlated with respiration rate variability. The inset shows the mean and standard deviation of correlation coefficients (connectivity) between the BOLD time series of the DM.LP_L and SA.SMG_R regions. See Table 6 for details.

Altered LCov connectivity was detected with three statistical tests:

- The test for different LCov connectivity to HC between pairs of 20 ROIs was positive for two ROI pairs (Table 4 and Figure 1). The brainstem medulla was involved in both, one connecting with the midbrain, the other with a DM hub. Both connections were *stronger* in LCov than HC.
- The test of connections between each of the 20 brain ROIs and all voxels in the rest of the brain identified clusters of voxels for which ROI correlations were different in LCov from HC. Table 5 lists the ROI seeds and cluster locations. Voxel clusters with different connectivity in LCov were detected for five SA and three DM network hubs (seeds). Most, but not all, cluster correlations were weaker in LCov than HC. Seven clusters were significant with p-FDR < 0.0001. The strongest statistical inference was seen for the SA.AI_L seed (p-FDR < 1e-6) for two clusters, one in the left temporal and one in the left frontal lobe (see Figure 2A). A cluster in the right operculum also correlated with the SA.AI_L region (Figure 2B) but with stronger connectivity in LCov than HC.
- The test for regressions in LCov between ROI-to-ROI connectivity and clinical scores revealed LC connectivity correlated with the disability score and two autonomic scores (Table 6 and Figure 3). A brainstem ROI (Mdul_R, PAG) was involved in two of the four significant correlations, and three involved salience network hubs (ACC, rPFC_L and rPFC_R). Because HC regressions with these clinical scores were insignificant, the LCov correlations can be regarded as abnormal. Two of the three clinical scores, HRV and RespV from data acquired during the scan, related to autonomic regulation.

**Table 4:** Two ROI pairs with reduced connectivity in 13 HC versus 10 LCov.

ROI1	ROI2	T(20)	p-FDR
Mdul_L	CnF_R	−4.22	0.0080
Mdul_L	DM.LP_R	−3.17	0.046

T is the T statistic. Negative T indicates weaker connectivity in HC. p-FDR is false discovery rate corrected statistical inference. ROI names are expanded in Table 1.

**Table 5:** ROI to voxel-cluster pairs with LCov connectivity different to HC.

ROI seed	LCov	Cluster location*	x y z	Cluster p-FDR and size (k)			
			(MNI)	0.001	k	0.005†	k
SA.AI_L	↓	infTemp/(occip part) L	−58 −38 −16	<1e-6	142	<1e-6	327
	↓	midFron L	−40 + 6 + 46	0.0060	34	<1e-6	311
	↓	precentral WM L	−32 0 + 36	0.0060	34	^	^
	↓	midFron L	−38 + 26 + 36	0.0060	34	^	^
	↑	Central Operculum R	+46–4 + 11			0.0001	152
SA.AI_R	↓	midTemp L	−54 −46 −14			0.00042	139
	↓	inf/midTemp Occip R	+56–44 −12			0.00072	118
SA.rPFC_R	↓	postCentral L	−38 −40 + 60	0.0081	40	0.000004	211
	↓	supraMarginal L	−44 −36 + 40	0.022	30	^	^
	↓	postCentral, SMG R	+40–26 + 36			0.0044	102
SA.SMG_L	↓	midtemp L	−64 −54 + 4	0.034	32	0.021	71
	↓	centralOperculum R	+58–6 + 12			0.021	76
	↓	midFron R	+34 + 4 + 58	0.034	25	0.047	58
SA.SMG_R	↑	latOccipInf&sup L	−38 −86 + 16			0.00007	158
	↑	supPar L	−28 −52 + 58			0.040	62
DM.PCC	↓	Angular L	−50 −56 + 28	0.00046	59	0.00009	158
	↓	midFron R	+36 + 14 + 42			0.036	66
DM.LP_L	↓	preCentral R	+48–18 + 58	0.0031	45	0.000002	227
	↑	midFron L	−42 + 36 + 26	0.0085	35	0.0069	88
DM.LP_R	↓	latOccip sup L	−38 −64 + 48	0.041	32	0.000002	237
	↓	Fron pole L	−42 + 54 + 4			0.0046	94
Mdul_R	↑	latOccip pole L	−28 −84 + 8			0.0035	89
HippoL	↓	Fron pole	+10 + 68 + 14	0.020	35	0.020	73
HippoR	↓	ParietalOperculum L	−42 −30 + 20	0.0099	36	0.020	65
	↓	paraCing supFron R	+4 + 46 + 20			0.0014	107
	↓	infTemp L	−60 −22 −26			0.020	62
Average 7	↓	midFron R	+56 + 14 + 40			0.011	78
Brainstem	↑	midFron / Fron pole R	+30 + 34 + 46			0.0060	85
Average 7SA	↓	Temp (occip part) L	−56 −48 −12			0.00056	138
Average 4DM	↓	Angular gyrus L	−50 −56 + 28			0.00015	158

ROI names are expanded in Table 1. The ‘LCov’ column indicates LCov connectivity is weaker (↓) or stronger (↑) than HC. Cluster statistics (p-FDR) and size (k voxels) for uncorrected voxel thresholds uvP < 0.001, and uvP < 0.005 are listed. Voxels were 1.25 mm isotropic.

* Temp, temporal; Occip, occipital; Fron, frontal; Cing, cingulate; ant, anterior; inf, inferior; mid, middle.

† Clusters separated with threshold uvP = 0.001 which merged with threshold 0.005 are indicated by ‘^’ below their parent cluster.

**Table 6:** ROI pairs for which Long Covid connectivity correlated with the clinical scores: bell disability, heart rate variability and respiration rate variability.

ROI1	ROI2	T(19)	p-FDR
Bell disability			
SA.ACC	SA.rPFC_L	+5.86	0.039
Heart rate variability			
PAG	Hippo_R	+4.87	0.034
Respiration rate variability			
Mdul_R	SA.rPFC_R	+5.64	0.015
DM.LP_L	SA.SMG_R	−5.59	0.016

T is the T statistic, p-FDR is the false discovery rate corrected statistical inference. The sign of T indicates whether the correlation was positive (+) or negative (−). ROI names are expanded in Table 1.

## 4. Discussion

In this pilot study we derived BOLD time series for 10 Long Covid and 13 Healthy Controls in 20 brain ROIS. Correlations between pairs of BOLD time series quantified connectivity between their ROIs. The ROIs included Salience (SA) and Default Mode (DM) Network hubs and subcortical ROIs (Table 1). Despite the small subject numbers, we showed several differences in LCov intrinsic brain connectivity relative to HC. Differences involving nuclei of the brainstem reticular activation system (RAS) suggest this regulatory structure may be pivotal to LCov symptoms.

### 4.1. LCov vs. HC comparisons – ROI to ROI connectivity

The principal finding was *increased* LCov connectivity relative to HC in the brainstem between the rostral medulla (Mdul_L) and midbrain cuneiform nucleus (CnF_R). There was also an increase from the Mdul_L to a DM hub (DM.LP_R). The first result is consistent with a brainstem volume study (Thapaliya et al., 2023) of the same subjects that reported *larger* volumes in LCov than HC. Both Mdul_L and CnF_R ROIs contain nuclei of the reticular activation system (RAS), an interconnected complex of small regulatory nuclei in the brainstem that influence cortical activity by both neurotransmitter release and oscillatory electrical stimulation. The midbrain CnF is located in the brainstem excitatory area and the rostral medulla in the brainstem inhibitory area (Hall, 2011). The ‘circuit’ between the two determines cortical excitation and sleep. In concert with the hypothalamus this circuit also controls autonomic function (Hall, 2011).

The excitatory CnF nuclei receive feedback via sensory signals from the periphery and signals from the cortex dependent on levels of brain thought or motor processes. Increases in physical or cortical activity then stimulate the excitatory area to maintain or enhance cortical activity (Hall, 2011). The connection from the medulla inhibitory area reduces excitatory output to facilitate the sleep–wake cycle.

*Increased* LCov connectivity in this circuit is perhaps counter-intuitive, and may be interpretated as an upregulation in RAS BOLD activity

- as a response to viral damage in the brainstem. Reported reactive gliosis and microglial activation (Pajo et al., 2021) may represent an injury that requires enhanced BOLD levels for normal brainstem connectivity.
- to compensate for broadly compromised cortical dysfunction (Fisher et al., 2021) and consequent reduced cortical feedback. The mechanism for this is unknown but may resemble the recently discovered regulatory process that maintains somatosensory performance in HC (Barnden et al., 2022).

The complexity of the RAS makes it difficult to exactly specify the RAS nuclei that generate the Mdul and CnF BOLD activity, although the Mdul ROI encompasses median raphe, gigantocellular and hypoglossal nuclei (Naidich et al., 2009), while the CnF involves in-part the midbrain reticular formation (MRF), pedunculotegmental nucleus and the pontis oralis (Athinoula, n.d.). The pedunculotegmental, also known as pedunculopontine (PPN), nucleus has been identified as the principal brainstem nucleus for activation of higher cortical regions (Garcia-Rill et al., 2016a,b, 2019).

The stronger Mdul_L to DM.LP_R connection in LCov is consistent with a relationship whereby increased Mdul_L activity in LCov decreases DM activity to facilitate executive network activity (Menon, 2011).

### 4.2. LCOV vs. HC comparisons – ROI to voxel connectivity

Involvement of brain regions beyond the 20 *a-priori* ROIs can be detected by tests for different LCov and HC connectivity between each ROI and the whole brain using voxel-based analysis. Table 5 lists multiple new regions thus detected. Differences were found brain areas connecting to 5 SA and 3 DM hubs, most but not all with HC > LCov. The middle frontal gyrus (bilaterally) and inferior and middle temporal lobes (left only) showed different LCov connectivity with SA hubs. Cluster defining thresholds of both uncorrected voxel *p* = 0.001 and 0.005 were examined. We felt the more liberal 0.005 threshold was justified because of the small voxel size and corresponding increase in BOLD time-series noise.

### 4.3. Connectivity regressions with LCov clinical scores

Regressions with disability and the autonomic measures of respiration rate variability and heart rate variability were significant for the brainstem Mdul_R and PAG in LCov but not HC, suggesting abnormal function of the RAS nuclei in those locations.

### 4.4. Stroop task performance

Long-Covid subjects performed the Stroop task with lower accuracy and slower reaction times than HC. Accuracy for the 3 categories of task was highest for the ‘neutral’ task in HC but lowest in LCov. This may indicate failure of the LCov subjects to understand or remember the instructions given for this category.

### 4.5. Comparison with ME/CFS

An ME/CFS fMRI study optimized for the brainstem (Barnden et al., 2019) reported *weaker* connectivity between the medulla and midbrain during a Stroop task. Here in LCov during the same task, medulla to midbrain connectivity was *stronger*. Thus, increased brainstem connectivity in LCov discriminates LCov from ME/CFS, although brainstem involvement is important in both. Larger sample sizes are required to clarify this. Duration of illness may be an important determinant for connectivity. The relatively acute (<2 years) symptoms of LCov compared to several years or decades for ME/CFS may be relevant. Longitudinal studies of LCov are needed to test for an evolution of brain pathology that may yield a pattern more like ME/CFS.

### 4.6. Potential biological mechanism

A recent investigation of impaired cell membrane calcium transport and transient receptor potential melastatin 3 (TRPM3) reported dysfunction in both ME/CFS and LCov (Cabanas et al., 2018; Sasso et al., 2022). These TRPM3 ion channels are widely expressed in the cerebellum, dorsal midbrain, amygdala and medulla (Allen Institute, 2022) and are believed to be involved in a range of biological processes including memory formation and consolidation, thermoregulation, pain, inflammation, and coordination (Oberwinkler and Philipp, 2014). Further, the prevalence of TRPM receptors in microglia could result in increased recruitment with neurotoxic potential (Bezzi et al., 2001; Ifuku et al., 2014). Microglial density is 10 times greater in the medulla than in the cortex (Mittelbron et al., 2001) and may account for medulla involvement in both ME/CFS and LCov.

### 4.7. ROI selection

To avoid excessive multiple-comparison corrections for statistical inference, we limited our analysis to 20 regions: 7 SA hubs, 4 DM hubs, 6 brainstem regions, two hippocampus and a cerebellum region (Table 1). This choice was informed by ME/CFS experience (Barnden et al., 2016, 2019). Independent Component Analysis (ICA) may provide a more comprehensive survey of altered intra-brain connectivity in LCov.

### 4.8. Limitations

The limited number of subjects in this pilot study required a corresponding limit of the number of brain regions between which connectivity was assessed. These preliminary results must be confirmed with more subjects. Future analysis of connectivity changes within and between the sequential 7.5 min runs in larger LCov cohorts will permit evaluation of more regions and may provide further insights into the rate of development of cognitive fatigue.

## 5. Conclusion

In this pilot study of brain connectivity in Long Covid, despite limited subject numbers, we have detected significant differences from HC, mostly in brainstem and salience network connections that are important for the regulation of brain function. Altered regulatory connections can have complex consequences that may manifest as the symptoms of LCov. In the brainstem during the same cognitive exertion, *enhanced* connectivity in LCov was opposite to the *impaired* connectivity in ME/CFS.

## Data availability statement

The datasets presented in this article are not readily available because datasets analyzed and/or generated during the current study are not publicly available due to confidentiality agreements. Requests to access the datasets should be directed to ncned@griffith.edu.au.

## Ethics statement

The studies involving human participants were reviewed and approved by the Griffith University Human Research Ethics Committee (2022/666). The patients/participants provided their written informed consent to participate in this study.

## Author contributions

LB: conceptualization and formal analysis. LB, KT, NE-F, and MB: methodology. LB and NE-F: writing of original draft. KT and SM-G: writing – review and editing. All authors contributed to the article and approved the submitted version.

## Funding

This research was funded by ME Research UK (MERUK, https://www.meresearch.org.uk/). Other funding bodies include: the Stafford Fox Medical Research Foundation (489798), the National Health and Medical Research Council (1199502), McCusker Charitable Foundation (49979), Ian and Talei Stewart, Buxton Foundation (4676), Henty Community (4879), Henty Lions Club (4880), Mason Foundation (47107), Mr. Douglas Stutt, Blake Beckett Trust Foundation (4579), Alison Hunter Memorial Foundation (4570), and the Change for ME Charity (4575).

## Conflict of interest

The authors declare that the research was conducted in the absence of any commercial or financial relationships that could be construed as a potential conflict of interest.

## Publisher’s note

All claims expressed in this article are solely those of the authors and do not necessarily represent those of their affiliated organizations, or those of the publisher, the editors and the reviewers. Any product that may be evaluated in this article, or claim that may be made by its manufacturer, is not guaranteed or endorsed by the publisher.

## Abbreviations

LCov: Long Covid
HC: Healthy Control
ROI: Region of Interest
ME/CFS: Myalgic Encephalomyelitis/Chronic Fatigue Syndrome
SA: Salience Network
DM: Default Mode Network
RAS: Reticular Activation System
